# Supplementary material for: Content-rich biological network constructed by mining PubMed abstracts
Source: BMC Bioinformatics. 2004 Oct 8;5:147. doi: 10.1186/1471-2105-5-147 (PMC528731; doi:10.1186/1471-2105-5-147)
Supplement: Additional File 2 — The original results of the above study (non-essential files are deleted to keep the file size under the limit set by BMC bioinformatics). [file 1471-2105-5-147-S2.bz2 › chilibotAdditionalFile2/dip05/70ID2966143E270/html/RECB_ABC2.html]

 


 **RECB** and **ABC2** 
  
Found 2 abstracts in PubMed, retrieved 2.  
 

 What does Google say? 
 PDF only 
| .edu only 

---

**Interactive relationship** (e.g. stimulation, inhibition, etc)

**Non-interactive relationship** (e.g. studied together, co-existance, homology, etc.)

- Overproduction of the RecC subunit in vivo, but not  **RecB**  or RecD, interfered with  **Abc2**  induced UV sensitization, revealing that RecC is the target for  **Abc2**  in vivo.  Ref: 10669596 J Mol Biol, 2000
- UV induced ATP crosslinking experiments revealed that  **Abc2**  protein does not interfere with the binding of ATP to either the  **RecB**  or RecD subunits in the absence of DNA, though it partially inhibits RecBCD ATPase activity.  Ref: 10669596 J Mol Biol, 2000
